# Cytokine levels as biomarkers of radiation fibrosis in patients treated with breast radiotherapy

**DOI:** 10.1186/1748-717X-9-103

**Published:** 2014-04-30

**Authors:** Charlotte B Westbury, Joanne Haviland, Sue Davies, Lone Gothard, Bahja Ahmed Abdi, Mark Sydenham, Jo Bowen, Richard Stratton, Susan C Short, John R Yarnold

**Affiliations:** 1 Department of Oncology, UCL Cancer Institute, University College London, London, UK; 2 Department of Oncology, Hillingdon Hospital, Middlesex, UK; 3 Mount Vernon Cancer Centre, Northwood, Middlesex, UK; 4 Institute of Cancer Research-Clinical Trials and Statistics Unit (ICR-CTSU), Institute of Cancer Research, Sutton, Surrey, UK; 5 Division of Radiotherapy and Imaging, Institute of Cancer Research, London and The Royal Marsden NHS Foundation Trust, Sutton, UK; 6 Centre for Rheumatology and Connective Tissue Disease, Royal Free and UCL Medical School, London, UK; 7 Cheltenham General Hospital, Cheltenham, UK; 8 Leeds Institute of Cancer and Pathology, University of Leeds, Leeds, UK

**Keywords:** Radiation fibrosis, Breast cancer, Biomarker, Interleukin-6, Connective tissue growth factor

## Abstract

**Background:**

Radiation fibrosis is not easily measurable although clinical scores have been developed for this purpose. Biomarkers present an alternative more objective approach to quantification, and estimation in blood provides accessible samples. We investigated if blood cytokines could be used to measure established fibrosis in patients who have undergone radiotherapy for breast cancer.

**Methods:**

We studied two cohorts treated by breast-conserving surgery and radiotherapy in the UK START Trial A, one with breast fibrosis (cases) and one with no or minimal fibrosis (controls). Two candidate cytokines, plasma connective tissue growth factor (CTGF) and serum interleukin-6 (IL6) were estimated by ELISA. Comparisons between cases and controls used the *t*-test or Mann–Whitney test and associations between blood concentration and clinical factors were assessed using the Spearman rank correlation coefficient.

**Results:**

Seventy patients were included (26 cases, 44 controls). Mean time since radiotherapy was 9.9 years (range 8.3-12.0). No statistically significant differences between cases and controls in serum IL6 (median (IQR) 0.84 pg/ml (0.57-1.14), 0.75 pg/ml (0.41-1.43) respectively) or plasma CTGF (331.4 pg/ml (234.8-602.9), 334.5 pg/ml (270.0-452.8) were identified. There were no significant associations between blood cytokine concentration and age, fibrosis severity, breast size or time since radiotherapy.

**Conclusions:**

No significant difference in IL6 or CTGF concentrations was detected between patients with breast fibrosis and controls with minimal or no fibrosis.

## Background

Radiation fibrosis is a component of the clinical spectrum of late radiation injury occurring after curative radiotherapy for cancer and is considered to be an important underlying cause of clinical morbidity. Radiation fibrosis is not easily measurable or quantifiable in the clinic which is one factor limiting the testing of effective therapeutic strategies. The current standard for measuring adverse effects of radiotherapy, including fibrosis, is by clinical score such as Late Effects Normal Tissues — Subjective, Objective, Management, Analytic (LENT SOMA) scale [1]. However this type of clinical score often encompasses complex functional endpoints [2], for which a number of underlying and uncertain pathologies may coexist. Even a simple biological endpoint such as fibrosis may not have direct clinical correlate, for example although breast induration is assumed to be due to fibrosis, oedema may be contributory. Alternative methods which may provide a more direct and precise estimate of fibrotic disease, include radiological imaging or the use of biomarkers, such as the estimation of cytokines in blood. Biomarker assessment can also be used to relate clinical and molecular responses, which may inform about the relevant molecular pathways involved in therapeutic response [3]. Tissue is not easily accessible, and measurement in blood provides a potential non-invasive assessment as a substitute to tissue analysis. In the context of radiation fibrosis, blood cytokines have been estimated to determine patterns of cytokine release in relation to clinical radiotherapy [4] or as a predictive test of lung toxicity after radiotherapy [5]. There are no reported studies investigating blood cytokines as biomarkers of established radiation fibrosis, which would provide a tool for quantifying fibrosis.

Following radiotherapy for breast cancer, gene expression changes at mRNA level can be detected in samples taken months or years after the treatment course in blood from patients with established breast fibrosis [6], and in breast tissue [7]. Genes identified in these studies include those encoding pro-inflammatory and pro-fibrotic cytokines. The findings of chronic alteration of gene expression support the hypothesis that blood cytokines implicated in radiation fibrosis could be effective biomarkers of radiation fibrosis. We selected two candidate biomarkers to determine as proof of principle whether they are altered in patients with radiation fibrosis. Interleukin 6 (IL6) and connective tissue growth factor (CTGF) were selected, as both were identified by gene expression analysis of breast tissue [7], and are implicated in the pathogenesis of radiation fibrosis. Furthermore IL6 and CTGF have shown roles as biomarkers in fibrotic disease of other aetiologies, including systemic sclerosis, idiopathic pulmonary fibrosis and liver fibrosis [8-11]. We compared blood cytokine levels in patients with established breast fibrosis compared to control cases with minimal or no fibrosis. Patients were identified from the UK START Trial A of breast radiotherapy in which comprehensive prospectively collected normal tissue toxicity data was available [12].

## Methods

### Ethical approval and patient selection

The UK START Trial A investigated different radiotherapy fractionation schemes in patients receiving radiotherapy for early breast cancer. Outcome measures for late normal tissue injury were prospectively recorded up to 10 years following radiotherapy using a validated clinical scoring system [13,14]. Scoring was performed using two criteria to assess fibrosis, annual clinical palpation to assess induration, and change in photographic breast appearance from baseline at 2 and 5 years (photographic scores). The photographic score was an indirect measure of fibrosis, assessing change in breast size and shape mainly due to shrinkage and distortion.

For the purpose of the current study, 2 groups of patients defined as cases with fibrosis and controls with minimal or no fibrosis, were selected from surviving START Trial A patients who had undergone previous breast conserving surgery and radiotherapy at The Royal Marsden (RM, Sutton), the Gloucestershire Oncology Centre (GOC, Cheltenham) or the Royal Berkshire Hospital (Reading) at least 5 years previously. Patient recruitment for the current study was planned primarily to be at RM, but was extended to include the Royal Berkshire Hospital and GOC (5 patients from the Royal Berkshire Hospital were referred and registered at RM for the purposes of this study). Patients with clinical and photographic scores at 2 years and beyond were included. Patients with fibrosis were defined as having moderate (‘quite a bit’) or marked (‘very much’) palpable induration at any time point between year 2 and the date last seen in clinic and some (mild or marked) change in photographic breast appearance at 2 or 5 years after radiation. Control patients had no or minimal (‘a little’) palpable induration at any time points from year 2 onwards and no change in photographic breast appearance at 2 and 5 years after radiation. Additional data available for all patients included measurements of breast length and maximum breast depth, recorded from the treatment plans on the START trial A radiotherapy form. Patients with locally recurrent or metastatic cancer and patients with a connective tissue disorder were excluded from the study. The study was approved by the UCLH Research Ethics Committee Alpha, under the protocol ‘Novel clinical biomarkers for the assessment of late normal tissue radiation fibrosis’, and all patients gave written informed consent to take part.

### Blood collection

Patients attended the RM, Sutton or GOC, Cheltenham on one occasion for venepuncture. Plasma and serum were obtained using standard protocols, centrifuged within one hour, and specimens were snap frozen immediately after centrifugation and stored at −80°C for future analysis. Measurement of CTGF in plasma and IL-6 in serum was carried out in accordance with previous reports and the manufacturer’s instructions for sample preparation for ELISA [11,15].

### ELISA

Samples were analysed blinded to case control status. Blood concentrations of IL6 and CTGF were determined with commercially available enzyme-linked immunosorbent assay (ELISA) kits, using a sandwich ELISA technique. IL6 concentrations were determined in serum (Quantikine HS, R&D Systems, Minneapolis) and CTGF in plasma (USCN, Life Science Inc, Wuhan). Assays were performed according to manufacturer’s instructions. The sensitivities of each assay were 0.039 pg/mL for serum IL6 and and 21 pg/ml for CTGF.

### Power calculations and statistical methods

A difference of 10 units in the mean blood concentrations of IL6 and CTGF between cases with fibrosis and controls with minimal or no fibrosis was considered to be of clinical importance. Assuming a standard deviation of 12.5 units, and with a 5% significance level and 80% power, it was calculated that 20 cases and 40 controls would be required. Patient and treatment characteristics were compared between cases and controls using the *t*-test for continuous variables and the chi-squared test or Fisher’s exact test for categorical variables. The distribution for CTGF was near normal so the *t*-test was used to compare CTGF levels. The distribution of IL6 concentration was skewed, so medians with interquartile ranges (IQR) and non-parametric methods were used. Comparisons between groups used the unpaired *t*-test or the non-parametric Mann–Whitney test as appropriate, and associations between quantitative variables were tested using Spearman’s rank correlation coefficient.

## Results

### Patient demographics

Of the 123 eligible patients invited to participate in the study, 72 consented. One case with a second primary breast cancer and one control patient with metastatic breast cancer were excluded as per protocol and specimens from the remaining 70 patients (26 cases and 44 controls) were analysed. Mean patient age at time of blood sampling was 65.8 years (SD 7.9, range 41.8-83.5), and mean time since radiotherapy was 9.9 years (SD 0.9, range 8.3-12.0). For the cases, the first recording of moderate or marked palpable induration was reported at a median of 4.4 years (IQR 3.0-6.4) following radiotherapy. Treatment details including the use of a radiotherapy boost, nodal irradiation, and adjuvant systemic therapy are shown in Table 1.

**Table 1:** Patient and treatment characteristics of cases and controls

	**Cases N = 26 (%)**	**Controls N = 44 (%)**	**Total N = 70 (%)**	**Comparison of cases & controls p-value**
**Age when blood sample taken**	66.3 (9.4)	65.5 (7.0)	65.8 (7.9)	0.720^2^
(years): Mean (SD)				
**Time since radiotherapy**	10.2 (1.2)	9.8 (0.8)	9.9 (0.9)	0.143^2^
(years): Mean (SD)				
**Radiotherapy schedule**				0.141
50 Gy in 25Fr	10 (38.5)	9 (20.4)	19 (27.1)	
41.6 Gy in 13Fr	11 (42.3)	18 (40.9)	29 (41.4)	
39 Gy in 13Fr	5 (19.2)	17 (38.6)	22 (31.4)	
**Tumour bed boost**				0.664^3^
No	3 (11.5)	3 (6.8)	6 (13.6)	
Yes	23 (88.5)	41 (93.2)	64 (91.4)	
**Lymphatic radiotherapy**				0.751^3,4^
None	21 (80.8)	37 (84.1)	58 (82.9)	
Axilla only	0 (0)	0 (0)	0 (0)	
SCF only	5 (19.2)	6 (13.6)	11 (15.7)	
Axilla and SCF	0 (0)	1 (2.3)	1 (1.4)	
**Adjuvant chemotherapy**				0.861
No	17 (65.4)	31 (70.4)	48 (65.6)	
Yes	9 (34.6)	13 (29.5)	22 (31.4)	
**Tamoxifen**				0.468^3^
No	2 (7.7)	7 (15.9)	9 (12.9)	
Yes	24 (92.3)	37 (84.1)	61 (87.1)	

SD = standard deviation.

Gy = Gray.

Fr = fractions.

SCF = supraclavicular fossa.

^1^p-value from chi-squared test unless otherwise indicated.

^2^p-value from *t*-test.

^3^Fisher’s exact test.

^4^chi-squared test of none versus any lymphatic radiotherapy.

### Interleukin 6

**Figure 1:**
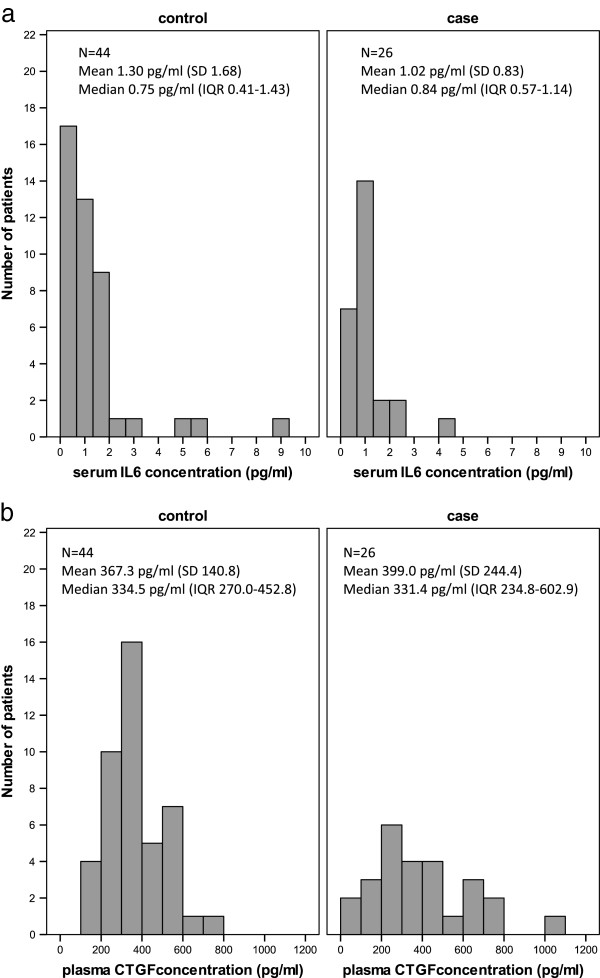
**Distributions of serum interleukin 6 and plasma connective tissue growth factor.** Distributions of serum IL6 **(a)** and plasma CTGF **(b)** in 26 cases with fibrosis and 44 controls without fibrosis selected from UK START Trial A patients (SD = standard deviation, IQR = interquartile range).

**Figure 2:**
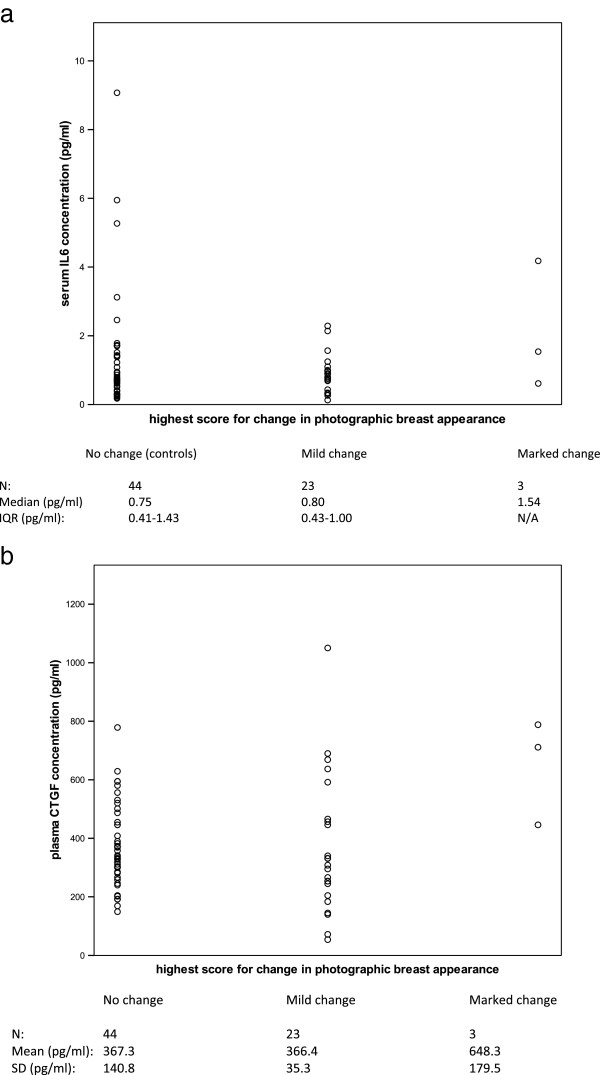
**Relationship of interleukin 6 and connective tissue growth factor to photographic score.** Distributions of **(a)** serum IL6 concentration and **(b)** plasma CTGF concentration according to highest score for change in photographic breast appearance at 2 or 5 years. In 70 patients selected from the UK START Trial A.

**Figure 3:**
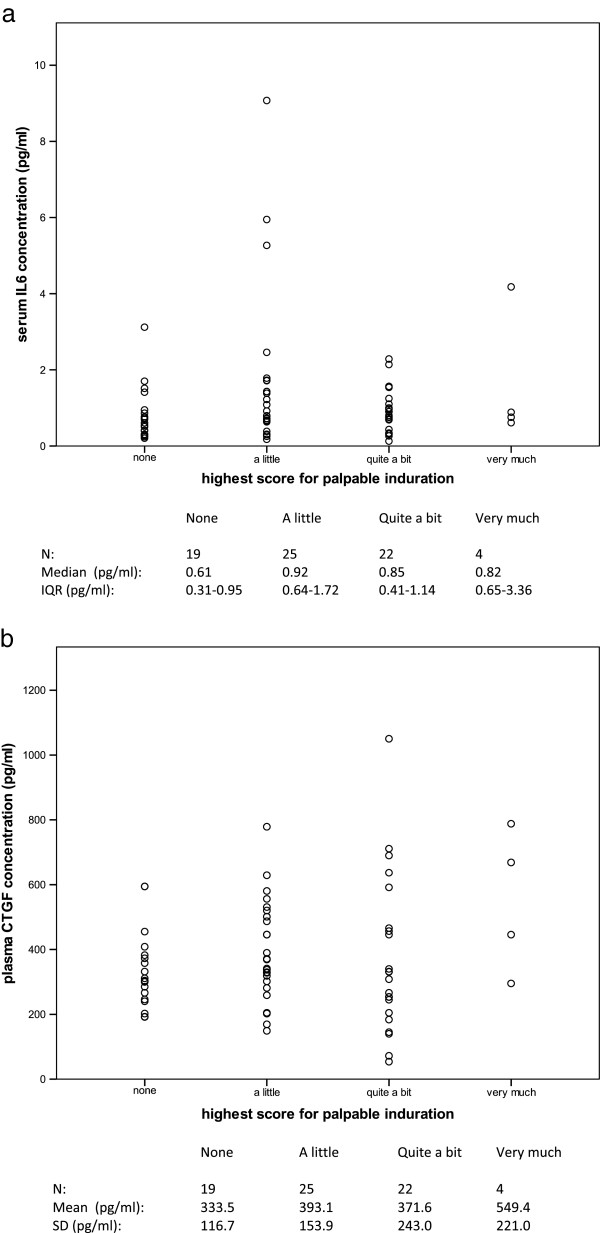
**Relationship of interleukin 6 and connective tissue growth factor to palpable induration.** Distributions of **(a)** serum IL6 concentration and **(b)** plasma CTGF concentration according to highest score for palpable induration beyond 2 years following radiotherapy. In 70 patients selected from the UK START Trial A.

Distributions of serum IL6 concentration in fibrosis cases and controls are shown in Figure 1a. Serum IL6 concentration was not statistically significantly different between cases and controls (Mann–Whitney test p = 0.771). There was no evidence of an association between serum IL6 concentration and age (Spearman correlation coefficient 0.15, p = 0.200), as has previously been reported [16]. The distributions of serum IL6 concentration according to highest photographic score recorded at 2 or 5 years are shown in Figure 2a; as only 3 cases with marked change in photographic breast appearance were recorded, no additional conclusions could be made for these cases (Spearman correlation coefficient 0.05, p = 0.654). There was no significant association between serum IL6 concentration and highest score for palpable induration (Figure 3a; Spearman correlation coefficient 0.14, p = 0.239).

There was no evidence of an association between serum IL6 concentration and volume of tissue irradiated, as shown by the Spearman correlation coefficients of 0.15 (p = 0.211) and 0.18 (p = 0.135) for breast length and maximum breast depth, respectively. There was no statistically significant association between serum IL6 concentration and time since radiotherapy treatment (Spearman correlation coefficient = 0.04, p = 0.719).

### CTGF

Distributions of plasma CTGF concentration in fibrosis cases and controls are shown in Figure 1b. There was no statistically significant difference in plasma CTGF concentration between cases and controls (*t*-test p = 0.550). The distribution of plasma CTGF according to highest photographic score recorded at 2 or 5 years is shown in Figure 2b; no additional conclusions could be made about a possible association due to the few cases with marked change in photographic breast appearance (Spearman correlation coefficient 0.04, p = 0.740). There was no significant association between plasma CTGF concentration and highest score for palpable induration (Figure 3b; Spearman correlation coefficient 0.10, p = 0.430).

There was no evidence of a significant association between plasma CTGF concentration and volume of breast irradiated as indicated by the Spearman correlation coefficients of 0.05 (p = 0.698) and 0.19 (p = 0.120) for breast length and maximum breast depth. There was no statistically significant association between plasma CTGF concentration and time since radiotherapy treatment (Spearman correlation coefficient = −0.04, p = 0.766).

## Discussion

This study was carried out to determine the potential utility of CTGF and IL6 concentrations in blood for evaluation of radiation fibrosis in a population of breast cancer patients with prospective clinical scores of fibrosis. Blood levels of these two candidate cytokines were not found to be significantly different between cases with fibrosis of the breast and controls with minimal or no fibrosis. This was a proof of principle study using a case control study design as one approach to investigating this question.

Two candidate cytokines were selected as possible biomarkers of radiation fibrosis, IL6 and CTGF. IL6, a proinflammatory cytokine expressed by a variety of cell types, contributes to endothelial cell dysfunction and stimulates leukocyte recruitment, as part of the vascular response to radiation. Vascular injury is considered to be an important factor contributing to radiation fibrosis although the exact mechanism by which it contributes to fibrogenesis is not known [17]. In human breast tissue previously treated with radiotherapy there is a chronic increase in expression of pro-fibrotic cytokines and up-regulation of networks and canonical pathways involved in inflammation and oxidative stress, including IL6 signalling [7]. CTGF has been extensively studied in fibrotic disease, including radiation fibrosis. It is an important growth factor in pro-fibrotic mechanisms including extracellular matrix production, and acts downstream of the well-characterised pro-fibrotic mediator transforming growth factor beta. CTGF is highly expressed in human tissues in chronic fibrotic states, including radiation enteritis and in the stroma of irradiated human breast [7,18]. Both IL6 and CTGF are proteins detectable in blood at increased levels in chronic inflammatory and fibrotic disease.

In this study, no relationship was found between cytokine level and volume of breast tissue irradiated. Tissue volume treated may be expected to influence the magnitude of cytokine response but the variation in measurements of breast volume may be too small to contribute to any detectable change in values of blood cytokine levels. For the purpose of the START Trial A, two-dimensional measurements of lung and heart (although not chest wall) incorporated into the radiotherapy treatment field were recorded. It is recognized that the pro-fibrotic response in non-target tissues (organs at risk) could be confounding, and clinical measurement of fibrosis in these tissues was not carried out. Examining lung length and maximum lung depth encompassed by the breast tangential fields, we did not show a significant correlation with cytokine levels (data not shown), although the increased tissue volume treated in the small proportion of patients who received nodal irradiation was not estimated.

Clinical scoring may be subjective and lack reproducibility. Of fifteen patients in the study who first scored moderate/marked for palpable induration before or at 5 years after radiotherapy, and who had follow-up data available beyond 5 years, only one third had a moderate/marked score after 5 years, suggesting limitations of reproducibility of clinical measurement (data not shown). With the likely complexity of underlying pathologies relating to the defined clinical endpoints ‘induration’ and ‘change in photographic breast appearance’, it is uncertain if these clinical endpoints accurately estimate actual fibrosis in the underlying tissue. Induration scored at less than 5 years could have included oedema rather than fibrosis alone.

This was a pragmatic approach to investigate the use of blood cytokines in radiation fibrosis, using an existing database of patients enrolled within a clinical trial with prospectively collected late toxicity scores. As information on possible confounding factors which may influence cytokine levels such as ethnicity and diet, or drugs such as statins and ACE inhibitors which may modulate fibrosis was not available in the database, we cannot exclude that these and other confounding factors exist. We did not include a control group with no previous radiotherapy in this study given the constraints on number of experiments and feasibility.

## Conclusions

This study shows that patients with clinically scored breast fibrosis have no difference in blood cytokine levels compared to patients with no or minimal fibrosis, after breast radiotherapy. A prospective longitudinal study correlating clinical scores of normal tissue toxicity and serial blood levels of cytokines after radiotherapy, including a baseline measurement, may be a more useful approach to establish the role of cytokines as biomarkers in radiation fibrosis. Furthermore, an approach using cytokine arrays to measure expression of multiple, non-candidate genes could provide more insight into the pathogenesis of fibrosis or mechanistic information about therapy response in future interventional studies where tissue is not easily accessible to biopsy.

## Abbreviations

IL6: Interleukin 6; CTGF: Connective tissue growth factor; ELISA: Enzyme-linked immunosorbent assay; SD: Standard deviation; IQR: Interquartile range.

## Competing interests

The authors declare that they have no competing interests.

## Authors’ contributions

CBW conceived and designed the study, carried out the assays for interleukin 6 and drafted the manuscript. JH contributed to the design of the study, performed the statistical analysis and helped draft the manuscript. SD and LG participated in study design and coordination and drafting of the manuscript. BAA carried out the assays for connective tissue growth factor. MS contributed to the data handling and helped draft the manuscript. JB participated in coordination of the study. RS contributed to study design and coordination. SS contributed to study design and drafting of the manuscript. JY designed the UK START Trials, contributed to current study design and helped draft the manuscript. All authors read and approved the final manuscript.
